# Use of Low-Cost Particle Counters for Cotton Dust Exposure Assessment in Textile Mills in Low- and Middle-Income Countries

**DOI:** 10.1093/annweh/wxab102

**Published:** 2021-11-14

**Authors:** Asaad Ahmed Nafees, Abdul Rehman Iqbal, Paul Cullinan, Sara De Matteis, Peter Burney, Sean Semple

**Affiliations:** Department of Community Health Sciences, Aga Khan University, Karachi, Pakistan; National Heart and Lung Institute (NHLI), Imperial College London, London, UK; Department of Community Health Sciences, Aga Khan University, Karachi, Pakistan; National Heart and Lung Institute (NHLI), Imperial College London, London, UK; National Heart and Lung Institute (NHLI), Imperial College London, London, UK; Department of Medical Sciences and Public Health, University of Cagliari, Cagliari, Italy; National Heart and Lung Institute (NHLI), Imperial College London, London, UK; Institute for Social Marketing and Health Research, University of Stirling, Stirling, Scotland, UK

**Keywords:** cotton fibres, textile industry, exposure assessment, dust, developing countries

## Abstract

**Objective:**

There is a lack of consensus on methods for cotton dust measurement in the textile industry, and techniques vary between countries—relying mostly on cumbersome, traditional approaches. We undertook comparisons of standard, gravimetric methods with low-cost optical particle counters for personal and area dust measurements in textile mills in Pakistan.

**Methods:**

We included male textile workers from the weaving sections of seven cotton mills in Karachi. We used the Institute of Occupational Medicine (IOM) sampler with a Casella Apex 2 standard pump and the Purple Air (PA-II-SD) for measuring personal exposures to inhalable airborne particles (*n* = 31). We used the Dylos DC1700 particle counter, in addition to the two above, for area-level measurements (*n* = 29).

**Results:**

There were no significant correlations between the IOM and PA for personal dust measurements using the original (*r* = −0.15, *P* = 0.4) or log-transformed data (*r* = −0.32, *P* = 0.07). Similarly, there were no significant correlations when comparing the IOM with either of the particle counters (PA and Dylos) for area dust measurements, using the original (*r* = −0.07, *P* = 0.7; *r* = 0.10, *P* = 0.6) or log-transformed data (*r* = −0.09, *P* = 0.6; *r* = 0.07, *P* = 0.7).

**Conclusion:**

Our findings show a lack of correlation between the gravimetric method and the use of particle counters in both personal and area measurements of cotton dust, precluding their use for measuring occupational exposures to airborne dust in textile mills. There continues to be a need to develop low-cost instruments to help textile industries in low- and middle-income countries to perform cotton dust exposure assessment.

**What’s Important About This Paper?**

There is a need to develop low-cost instruments to help textile industries in low- and middle-income countries undertake cotton dust exposure assessments. This study found that particle concentrations measured with two low-cost particle counters (Purple Air and Dylos) were not correlated with a standard method (IOM samplers with gravimetric analysis) in cotton mills in Karachi, Pakistan. These low-cost optical particle counters may not provide a satisfactory alternative to gravimetric methods of measuring occupational exposure to airborne dust in this setting.

## Introduction

Byssinosis is an occupational respiratory disease typically associated with exposure to cotton dust among textile workers. It develops progressively after prolonged exposure over several years (Schilling *et al.*, 1963) and is largely preventable by dust control measures in the workplace (NIOSH, 1986). There is a lack of consensus on methods for cotton dust measurement in the textile industry, and techniques vary between countries. In the UK, for example, standards are based on the use of the Institute of Occupational Medicine (IOM) sampling head (HSE, 2002a), whereas those in the US call for the use of vertical elutriators (OSHA, 1981). The particle size to be measured and permissible levels also vary across countries. This is despite the fact that health-based sampling principles have been well established and are generally recognized globally (ACGIH, 1985, ISO, 2012)—it seems that cotton dust sampling procedures have not been updated in some countries. In any case, the use of cumbersome instruments and lengthy procedures undermines the widespread use of exposure monitoring by environmental health and safety managers at textile mills, especially perhaps in resource-poor settings.

MultiTex is a randomized controlled trial of a low-cost, multi-component intervention to improve dust control and worker health in cotton textile mills in Karachi, Pakistan (Nafees *et al.*, 2019). As part of the study, we undertook comparisons of standard, gravimetric methods with low-cost optical particle counters for personal and area dust measurements in five mills.

## Methods

### Setting and population

Textile workers in Pakistan work in weekly shift patterns with 8- or 12-h shifts, depending on the type and size of mill. For each of these experiments, we included male textile workers from the weaving sections of five textile mills in Karachi.

### Dust measurement

We used the IOM sampler with a 25-mm MCE glass fibre filter for the collection of inhalable airborne particles. The sampler was attached to a Casella Apex 2 standard pump operating at 2 l min^−1^ and was clipped to workers’ collars. Such an arrangement allows the IOM sampler to trap particles up to 100 μm in aerodynamic diameter, within the breathing zone of workers; closely simulating the way particles are inhaled through the nose and mouth. The filters were pre- and post-weighed as a single unit; all particles collected were included in the analysis. For weighing, we used a fine weighing scale in a temperature and humidity-controlled environment; changes in weights were recorded in micrograms. We used one field blank for each batch of 10 filters.

The Purple Air (PA-II-SD) device is a wearable, air quality sensor that measures real-time PM_2.5_ concentrations. It uses a fan to draw air past a laser, causing reflections from dust particles that may be counted in sizes between 0.3 and 10 μm diameter. Using 1-s particle counts, estimated total mass for PM_2.5_ can be averaged using the device. Built-in Wi-Fi enables the sensor to upload readings to the cloud, and store in the PurpleAir map, from where data can be downloaded. PA has been used for measuring ambient air pollution in African countries (Awokola *et al.*, 2020).

The Dylos DC1700 is a static particle counter using laser beams to detect passing particles by their reflectivity. The sensors count particles in two sizes of >0.5 and >2.5 µm; the particle counts can be converted to PM_2.5_ mass in µg m^−3^ (Semple *et al.*, 2015).

### Experimental procedures

For experiment I, personal dust measurements were undertaken on 32 machine operators from the weaving sections of five textile mills. IOM and PA samplers were attached in parallel, on the same worker. The personal dust measurements were performed using a standard approach for gravimetric sampling (HSE, 2002b). For experiment II, we included 30 area dust measurements in the weaving sections of five textile mills. The IOM and PA samplers and Dylos monitors were placed adjacent to each other on a designated place near the centre of the section.

For both experiments, sampling was performed for 6 and 8 h for 8- and 12-h working shifts, respectively, during the daytime. Temperature and humidity were recorded at the workplace. Personal and area-level dust exposures were estimated by determining the 8-h time weighted average (TWA) for each worker.

### Statistical analysis

We discarded one sample each in experiments I and II due to inadequate duration of measurement; analyses were of 31 and 29 samples, respectively. We calculated the 8-h TWA values in µg m^−3^ for dust measurements carried out in each experiment, and report arithmetic means, and geometric means (GM) with standard deviations (GSD). We developed scatter plots and calculated Pearson coefficients for determining correlations between different instruments. We re-assessed the correlations after log transformation of data.

The study was approved by the ethics committees at Aga Khan University, Karachi (2019-0962-3710), the National Bioethics Committee in Pakistan (4-87/NBC-402/19/483), and Imperial College London (19IC4968).

## Results

**Figure 1.**
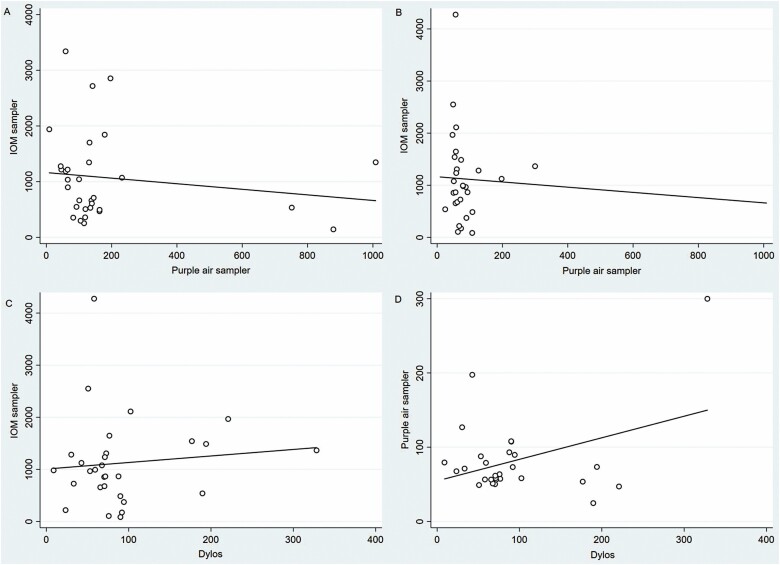
Scatter plots for experiment I (personal monitoring; *n* = 31) and II (area monitoring; *n* = 29), 8-h TWA (µg m^−3^). (A) IOM sampler and purple air sampler (experiment I). (B) IOM sampler and purple air sampler (experiment II). (C) IOM sampler and Dylos air quality monitor (experiment II). (D) Purple air sampler and Dylos air quality monitor (experiment II).

The overall GM (GSD) personal dust exposure obtained from IOM and PA for 31 participants in experiment I were 830.5 (±2.1) and 120.6 (±2.4) µg m^−3^, respectively (Table 1). There were no significant correlations between the two sets of measurements using the original (*r* = −0.15, *P* = 0.4) (Figure 1) or log-transformed data (*r* = −0.32, *P* = 0.07), including after removing outliers.

**Table 1.** Overall and mill-level personal and area dust concentration (8-h TWA, µg m^−3^) in experiments I and II^a^

Variable	Experiment I			Experiment II		
	n	AM	GM (GSD)	n	AM	GM (GSD)
Overall						
IOM	31	1069.9	830.5 (2.1)	29	1121.7	824.2 (2.5)
PA	31	186.7	120.6 (2.4)	29	81.3	71.8 (1.6)
Dylos	—	—	—	29	92.0	73.2 (2.0)
Mill A						
IOM	2	1578.3	1536.3 (1.4)	—	—	—
PA	2	27.6	20.6 (3.1)	—	—	—
Dylos	—	—	—	—	—	—
Mill B	—	—	—	—	—	—
IOM	—	—	—	1	1364.9	—
PA	—	—	—	1	299.9	—
Dylos	—	—	—	1	328.2	—
Mill C	—	—	—			
IOM	—	—	—	4	452.2	339.1 (2.7)
PA	—	—	—	4	99.6	99.2 (1.1)
Dylos	—	—	—	4	90.5	90.4 (1.0)
Mill D						
IOM	2	1104.4	928.9 (2.4)	2	900.4	872.3 (1.4)
PA	2	126.1	126.0 (1.1)	2	129.6	110.3 (2.3)
Dylos	—	—	—	2	56.6	54.9 (1.4)
Mill E						
IOM	19	1055.3	756.2 (2.3)	20	1223.3	921.4 (2.4)
PA	19	173.8	141.8 (1.7)	20	66.4	64.6 (1.3)
Dylos	—	—	—	20	81.2	65.1 (2.1)
Mill F						
IOM	2	1126.3	1122.6 (1.1)	2	1544.5	1172.2 (3.0)
PA	2	65.5	65.5 (1.0)	2	36.9	34.8 9 (1.6)
Dylos	—	—	—	2	120.2	98.2 (2.5)
Mill G						
IOM	6	916.18	792.7 (1.9)	—	—	—
PA	6	340.93	157.4 (3.9)	—	—	—
Dylos	—	—	—	—	—	—

AM, arithmetic means; GM, geometric means; IOM, Institute of Occupational Medicine; PA, Purple Air; TWA, time-weighted average.

^a^Experiment I undertook comparison of IOM and PA for personal dust measurements; experiment II considered comparison between IOM, PA, and Dylos for area dust measurements.

For experiment II, the overall, GM (±GSD) for area dust exposures obtained using the IOM, PA, and Dylos were, respectively, 824.2 (±2.5), 71.8 (±1.6), and 73.2 (±2) µg m^−3^ (Table 1). Again, there were no significant correlations when comparing the gravimetric method (IOM) with either of the particle counters (PA and Dylos) using the original (*r* = −0.07, *P* = 0.7; *r* = 0.10, *P* = 0.6) (Figure 1) or log-transformed data (*r* = −0.09, *P* = 0.6; *r* = 0.07, *P* = 0.7). There was a marginally significant correlation between measurements from the two particle counters when using raw data (*r* = 0.375, *P* = 0.045) (Figure 1). Findings were similar when we calculated the correlations after removing the outliers.

Using the IOM data in experiment I, we found higher exposures among weavers working on air-jet, compared with shuttle-less looms [GM ±GSD: 1020 µg m^−3^ (± 1.9) versus 624.6 µg m^−3^ (±2.2); *P* = 0.045]. We found higher exposure among those working at humidity levels ≤70% [1059.4 µg m^−3^ (±2) versus 617.9 µg m^−3^ (±2); *P* = 0.035]. These differences were not found in data from the PA (Supplementary Table 1).

Similarly, using the IOM data in experiment II, we found higher area exposures in weaving rooms using air-jet compared with shuttle-less looms [1150.1 µg m^−3^ (± 2.3) versus 576.7 µg m^−3^ (±2.3); *P* = 0.011]. We found a similar trend using the PA, but not the Dylos data (Supplementary Table 1).

## Discussion

Our findings show a lack of correlation between the gravimetric method and the use of particle counters in both personal and area measurements of cotton dust. The latter seem unlikely to be helpful in measuring dust concentrations in textile mills and cannot substitute for the traditional, more expensive approach.

Our findings may be explained in several ways. Cotton textile dust is likely comprised of high numbers of large particles, in comparison to combustion-derived aerosols where the particulate matter produced is generally below 2.5 µm in diameter: previous work using the optical particle counters to measure second-hand tobacco smoke or smoke from household cooking have shown good correlation between gravimetric and particle counters (Lim *et al.*, 2018, Coffey *et al.*, 2019). Several studies have shown that Dylos may be used as a simple low-cost substitute for gravimetric analysis when measuring fine particles, such as second-hand cigarette smoke or ambient air pollution (Semple *et al.*, 2015, Carvlin *et al.*, 2017, Ferdous *et al.*, 2020). Similarly, PA has been used to measure fine particle ambient air pollution in various settings (Mousavi *et al.*, 2021). Moreover, we found the use of the Dylos counter particularly problematic since larger cotton particles (‘fluff’) tended to choke the device’s internal fan, necessitating frequent cleaning during field sampling.

As far as we are aware, particle counting devices have only rarely been used to assess occupational exposures to dust comprising larger particles such as that common in textile mills. Recently, Khan *et al.* (2015) undertook a study involving 47 cotton factories in the Faisalabad region of Pakistan where they determined cotton dust exposures using particle counters (Grimm Portable Aerosol Spectrometer 1108, and the MiniDiSC), in addition to IOM samplers. Compared to our findings for area measurements (PA = 0.08 mg m^−3^, Dylos = 0.09 mg m^−3^), they reported a higher PM_2.5_ level, 0.57 mg m^−3^. Moreover, compared with our finding for gravimetric analysis (IOM; 1.07 mg m^−3^), they report a higher level of 2.55 mg m^−3^ for the inhalable fraction. They report too that, on average, over 50% of the total dust measured was from coarse particles (>2.5 µm) but also found a high level of correlation (*R*^2^ = 0.7–0.8) between fine and coarse particle concentrations, suggesting that instruments measuring PM_2.5_ could be used to reliably provide indications of inhalable dust concentrations. Our findings with the low-cost PA and Dylos devices do not replicate their findings with the GRIMM and MiniDiSC devices, perhaps reflecting the different operation of these higher cost instruments.

A potential limitation of our work is the fact that optical particle counters are generally manufactured to provide an estimate for the fine particles in the respirable fraction (≤PM_2.5_) and these may not be appropriate for comparison with the IOM samplers, designed to estimate the inhalable fraction (between PM_10_ and PM_100_). Recalibration of these devices by the manufacturers resulting in provision of another calibration curve, or a fixed factor across the whole concentration range could be a possible solution to this problem. Another limitation includes the fact that both the PA and Dylos make use of measurement principles that count particles in the air, such particulate counts may be biased due to physical properties of particles (like size and shape). Moreover, these samplers may need a regular calibration while being used—that was not done in our study.

## Conclusion

We conclude low-cost optical particle counters are not a satisfactory alternative to gravimetric methods for measuring occupational exposure to airborne dust in textile mills. There continues to be a need to develop low-cost instruments to help textile industries in low- and middle-income countries perform cotton dust measurement to aid in controlling workers’ exposure.

## Supplementary Material

wxab102_suppl_Supplementary_Table_1

## Data Availability

Data are available on reasonable request.
