# Supplementary material for: Use of Low-Cost Particle Counters for Cotton Dust Exposure Assessment in Textile Mills in Low- and Middle-Income Countries
Source: Ann Work Expo Health. 2021 Nov 14;66(4):537–42. doi: 10.1093/annweh/wxab102 (PMC9030129; doi:10.1093/annweh/wxab102)
Supplement: wxab102_suppl_Supplementary_Table_1 [file wxab102_suppl_supplementary_table_1.docx]

| Supplementary Table 1: Personal and area dust concentration (8-Hours TWA, µg/m^3^) according to occupational and mill-level factors in experiments I and II* | | | | | | | | | | | | | | | | | |
| --- | --- | --- | --- | --- | --- | --- | --- | --- | --- | --- | --- | --- | --- | --- | --- | --- | --- |
| Variables (n)$ | Experiment I | | | | | | | Experiment II | | | | | | | | | |
|  |  | IOM | | | PA | | |  | IOM | | | PA | | | Dylos | | |
|  | n | AM | GM (GSD) | P-value | AM | GM (GSD) | P-value | n | AM | GM (GSD) | P-value | AM | GM (GSD) | P-value | AM | GM (GSD) | P-value |
| Type of mill⁋ |  |  |  |  |  |  |  |  |  |  |  |  |  |  |  |  |  |
| Good mills (4; 3) | 12 | 1092.9 | 963.1 (1.8) | .29 | 207.0 | 93.4 (3.5.) | .03 | 8 | 837.3 | 585.6 (2.7) | .10 | 91.4 | 78.4 (1.9) | .24 | 89.5 | 81.5 (1.6) | .53 |
| Poor mills (1; 2) ** | 19 | 1055.3 | 756.2 (2.3) |  | 173.8 | 141.8 (1.7) |  | 21 | 1230.0 | 938.8 (2.4) |  | 77.5 | 69.5 (1.5) |  | 93.0 | 70.3 (2.2) |  |
| Shift duration |  |  |  |  |  |  |  |  |  |  |  |  |  |  |  |  |  |
| 8 hours (4; 3) | 12 | 1092.9 | 963.1 (1.8) | .29 | 207.0 | 93.4 (3.5) | .03 | 5 | 1250.9 | 1073.7 (1.8) | .60 | 126.6 | 84.9 (2.8) | 1.0 | 136.4 | 99.1 (2.4) | .81 |
| 12 hours (1; 2) ** | 19 | 1055.3 | 756.2 (2.3) |  | 173.8 | 141.8 (1.7) |  | 24 | 1094.7 | 780.0 (2.6) |  | 71.9 | 69.4 (1.3) |  | 82.7 | 68.7 (2.0) |  |
| Type of machines# |  |  |  |  |  |  |  |  |  |  |  |  |  |  |  |  |  |
| Air-jet (4; 2) | 18 | 1222.2 | 1020.1 (1.9) | .045 | 188.1 | 108.2 (2.8) | .496 | 15 | 1481.1 | 1150.1 (2.3) | .01 | 54.5 | 53.2 (1.3) | <0.001 | 104.3 | 92.1 (1.6) | .16 |
| Shuttle-less (2;4) | 13 | 859.0 | 624.6 (2.2) |  | 184.7 | 140.1 (1.9) |  | 14 | 736.6 | 576.7 (2.3) |  | 110.2 | 99.0 (1.5) |  | 78.8 | 57.2 (2.3) |  |
| Humidity |  |  |  |  |  |  |  |  |  |  |  |  |  |  |  |  |  |
| ≤ 70% | 17 | 1304.5 | 1059.4 (2.0) | .035 | 184.6 | 100.5 (2.9) | .057 | 14 | 1261.3 | 1067.6 (2.0) | .09 | 79.4 | 67.0 (1.7) | .38 | 110.9 | 80.4 (2.5) | .40 |
| > 70% | 14 | 785.0 | 617.9 (2.0) |  | 189.1 | 150.5 (1.8) |  | 15 | 990.9 | 647.3(2.8) |  | 83.2 | 76.7 (1.5) |  | 74.4 | 67.1 (1.6) |  |
| Temperature |  |  |  |  |  |  |  |  |  |  |  |  |  |  |  |  |  |
| ≤ 30^o^ C | 11 | 1136.3 | 937.5 (1.9) | .50 | 96.6 | 79.5 (2.2) | .137 | 19 | 1031.5 | 699.2(2.7) | .06 | 78.2 | 71.1 (1.6) | .81 | 79.5 | 71.5 (1.6) | .46 |
| > 30^o^ C | 20 | 1033.4 | 776.9 (2.2) |  | 236.2 | 151.7 (2.4) |  | 10 | 1293.0 | 1126.5(1.9) |  | 87.3 | 73.3 (1.7) |  | 115.7 | 76.6 (2.9) |  |
| *Experiment I undertook comparison of IOM and PA for personal dust measurements; experiment II considered comparison between IOM, PA and Dylos for area dust measurements  $number of mills included (experiment I; experiment II)  **There was only one poor mill in experiment I, which also had a shift duration of 12 hours.  ⁋ According to the occupational health and safety status of each mill, assessed using a checklist (Nafees et al., 2019).  #At one of the mills (E) both type of looms (air-jet and shuttle-less) were present and were considered separately from each other (hence number of mills is 6). | | | | | | | | | | | | | | | | | |

NAFEES, A. A., DE MATTEIS, S., KADIR, M. M., BURNEY, P., COGGON, D., SEMPLE, S. & CULLINAN, P. 2019. MultiTex RCT - a multifaceted intervention package for protection against cotton dust exposure among textile workers - a cluster randomized controlled trial in Pakistan: study protocol. *Trials,* 20**,** 722.
